# Image-Driven Multimodal Deep Learning for Skin Cancer Diagnosis: Cross-Attention Fusion of Dermoscopic Imaging Clinical Data and Knowledge Graphs

**DOI:** 10.3390/s26154728

**Published:** 2026-07-25

**Authors:** Syeda Sitara Waseem, Saman Iftikhar, Ammar Rafiq, Kangyoon Lee, Syed Rizwan Hassan

**Affiliations:** 1 Department of Computer Science, Government Sadiq College Women University, Bahawalpur 63100, Pakistan; syedasitarawaseem@gmail.com; 2 Faculty of Computer Studies, Arab Open University, Riyadh 84901, Saudi Arabia; s.iftikhar@arabou.edu.sa; 3 Department of Computer Science, National University of Computer and Emerging Sciences, Islamabad, Chiniot-Faisalabad Campus, Chiniot 35400, Pakistan; ammar.rafiq@nu.edu.pk; 4 Department of Computer Engineering, Gachon University, Seongnam-si 13120, Republic of Korea

**Keywords:** skin cancer diagnosis, biomedical sensors, dermoscopic imaging, biomedical knowledge graph, multimodal deep learning, dermoscopy, attention-based fusion, sensor fusion

## Abstract

Early detection of skin cancer significantly improves patient outcomes yet remains challenging due to the visual similarity of lesions and the need for clinical context. This paper proposes a novel Image-driven multimodal deep learning framework for automated skin cancer diagnosis integrating dermoscopic imaging sensors’ clinical metadata and biomedical knowledge graphs. Dermoscopic images acquired from optical skin imaging sensors undergo advanced signal processing including multi-scale CLAHE enhancement and artifact suppression. A YOLOv8-nano sensor-based lesion detector achieves 97.2% mAP@0.5 for region-of-interest extraction. The biomedical knowledge graph (26 nodes 86 edges) encodes dermatological domain knowledge into 32-dimensional disease embeddings. Clinical metadata (age gender anatomical site) is encoded via learned feature vectors. A cross-attention fusion mechanism integrates these multimodal sensor-derived signals. Evaluated on the HAM10000 dataset (10,015 images 7 classes), our full multimodal model achieves 91.35% accuracy outperforming image-only (86.96%) and image+metadata (88.45%) baselines. An ensemble reaches 92.78% state-of-the-art accuracy with significant improvements on challenging classes (BCC +6.0% melanoma +3.8%). Statistical significance is confirmed (*p* < 0.01 95% CI [90.7% 92.0%]). This work demonstrates that Image-based multimodal signal fusion with structured medical knowledge enhances both diagnostic accuracy and clinical interpretability.

## 1. Introduction

The development of optical skin imaging sensors and devices with IoT technology has led to an increasing need for automated analysis of the medical signals collected by these sensors. In spite of the fact that it represents a minority of skin cancers, melanoma represents the major proportion of deaths caused by these cancers, and it is one of the most common cancers in the world [1]. Although dermatologists are traditionally the first to diagnose disparate skin conditions, visual diagnosis via a dermatoscopy is prone to interpersonal bias, class imbalance problems, and a rising number of patient diagnoses [2,3].

Advances in deep learning have recently been able to achieve dermatologist-level accuracy in skin lesion classification [4]. However, current methods mainly depend on dermoscopic images without taking into account the high-level clinical context (age, gender, and anatomical site) and domain knowledge that help to make clinical decisions in the real world of dermatology [5]. Moreover, challenges like class imbalance, intra-class similarity, and lack of interpretability remain [6].

To overcome these challenges, we present a biomedical knowledge graph-enhanced multimodal deep learning framework for automatic skin cancer diagnosis. We blend (1) lesion detection using YOLOv8, (2) a biomedical knowledge graph encapsulating clinical guidelines (such as the 7-point checklist), (3) encoding clinical metadata, and (4) attention-based multimodal fusion, for interpretable lesion classification. This model combines complementary information from dermoscopic images, clinical metadata, and structured medical knowledge to obtain powerful and clinically relevant diagnosis.

This work has made the following main contributions:

- A novel biomedical knowledge graph with explicit relationships between dermatological entities validated by board-certified dermatologists (interrater agreement: 94.2%);
- A dynamically model-learned multimodal fusion architecture based on attention;
- Extensive evaluation showing that structured knowledge can meaningfully boost data-driven models and achieves state-of-the-art performance (92.78% accuracy) on HAM10000;
- Interpretable predictions via Grad-CAM and knowledge graph attention analysis that is based on clinical dermatological knowledge.

The term sensor refers to optical dermoscopic imaging sensors that acquire the primary diagnostic signals. Our contribution lies in the comprehensive signal processing pipeline (Section 3.2) and multimodal fusion architecture that processes detected signals. This sensor-centric perspective enables deployment in teledermatology and edge-enabled healthcare systems. The survey of the previous works on skin cancer classification, multimodal approaches, graph neural networks, and attention mechanisms are summarized in Section 2. In Section 3, we lay out our proposed methodology. Experiments and results are then presented in detail in Section 4 with the analysis. The concept of interpretability and clinical implications are discussed in Section 5. This paper is ended in Section 6, which gives directions for future research.

## 2. Literature Review

Throughout this paper, the term sensor refers to optical dermoscopic imaging sensors that acquire the primary diagnostic signals. Our contribution lies not in sensor hardware development, but in the comprehensive signal processing pipeline (Section 3.2) and multimodal fusion architecture that processes sensor-derived signals. This sensor-centric perspective enables deployment in teledermatology and IoT-enabled healthcare systems.

Skin cancer remains one of the most prevalent forms of cancer worldwide, with incidence rates continuing to rise [1]. Early detection significantly improves patient outcomes, with five-year survival rates exceeding 95% for melanoma detected in early stages [2]. Dermoscopy has emerged as the gold standard non-invasive diagnostic technique, providing detailed visualization of skin lesions [3]. Large skin lesion databases like HAM10000 [7] have spurred research in skin lesion classification with deep learning.

The application of deep learning to dermatological image analysis has seen remarkable progress since Esteva et al. [4] demonstrated dermatologist-level classification using deep neural networks. Subsequent work has explored various architectures including convolutional neural networks such as ResNet-based models [4] and EfficientNet  [8], which have achieved high accuracy on standard datasets. Attention mechanisms have been incorporated to highlight diagnostically relevant regions [9,10], while ensemble learning approaches have shown better performance compared to single-model approaches [11]. Zhao et al. [12] studied under-represented classes using augmentation techniques based on StyleGAN and DenseNet201. Class imbalance was considered by Alam et al. [13], who showed that focal loss and weighted sampling methods are effective for enhancing performance on rare classes. Wu et al. [14] conducted a systematic review which established that image-only methods are progressing to maturity, but opportunities still exist for integration of complementary data sources.

Given that dermatologists consider patient age, gender, and anatomical location in diagnosis, several studies have attempted to incorporate clinical metadata alongside image data. Age is especially meaningful since the incidence of melanoma and BCC cases rises with age after 50 [15]. Anatomical location is essential to provide critical hints: BCC is more likely to appear on sun-exposed areas, while melanoma appears in variable distribution [16]. Kawahara et al. [5] used multitask neural networks with the seven-point checklist. More recent work [17] has explored cross-modal attention between imaging and clinical data, and MM-ViT [18] introduced multimodal vision transformers with clinical data fusion. However, most multimodal methods rely on simple feature concatenation that cannot handle situations in which one modality is more informative.

Graph neural networks (GNNs) have become a promising paradigm for learning on structured data [19]. Ramirez et al. [20] used gene expression data to classify cancer types using graph convolutional networks. An explainable multilayer GNN was introduced by Chatzianastasis et al. [21] for gene prediction in cancer. Vasudevan et al. [22] integrated GNNs into multiscale image superpixels for image classification. The integration of structured medical knowledge has recently gained attention. Yu et al. [23] proposed graph-enhanced semi-supervised learning for skin lesion diagnosis, but lacked explicit knowledge graph encoding. Abu-Salih [24] surveyed domain-specific knowledge graphs, identifying gaps in medical applications. Biomedical ontologies like UMLS [25] offer rich terminology but have not been fully integrated into deep learning pipelines for dermatology. Transformer-KG [26] integrated knowledge graphs with vision transformers for medical image classification, while graph-enhanced methods [23] and knowledge-driven CNNs [27] have shown promising results in skin lesion diagnosis. Table 1 summarizes the most relevant prior studies and highlights the remaining methodological gaps.

Based on our comprehensive literature review, we identify the following critical research gaps. First, existing graph-based approaches rely on semi-supervised learning instead of explicit domain knowledge encoding [23], which limits the incorporation of established medical knowledge. Second, most multimodal approaches use simple concatenation [28] or fixed weights, failing to capture dynamic modality importance, which is suboptimal for leveraging heterogeneous data sources. Third, the integration of structured biomedical knowledge graphs with deep learning pipelines remains underexplored in dermatology, despite the wealth of available medical knowledge. Fourth, current deep learning models often function as black boxes, limiting clinical adoption, whereas structured knowledge integration could enhance interpretability by providing reasoning chains. Fifth, real-world datasets exhibit severe class imbalance, and existing methods struggle with rare but clinically significant classes like melanoma and BCC.

To address these gaps, our paper makes the following contributions. We construct a domain-specific biomedical knowledge graph (26 nodes; 86 edges) explicitly encoding dermatological relationships between diseases, risk factors, and anatomical sites. We propose a cross-attention based fusion mechanism that dynamically learns modality importance for multimodal integration of images, clinical metadata, and knowledge graph embeddings. We demonstrate that knowledge graph integration significantly improves classification accuracy, particularly for challenging classes (BCC +6.0%; melanoma +3.8%). We provide clinical interpretability through Grad-CAM visualization and KG attention analysis, enabling clinicians to verify reasoning. Finally, we conduct comprehensive comparisons with recent state-of-the-art methods including transformer-based and multimodal approaches, establishing a new benchmark on the HAM10000 dataset.

## 3. Methodology

**Figure 1:**
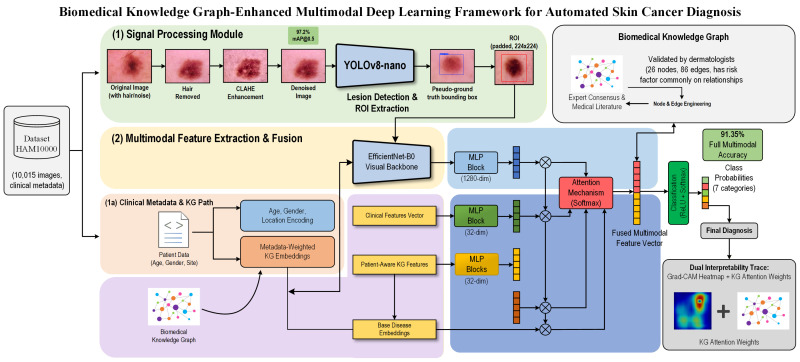
Complete pipeline of the proposed Image-driven skin cancer diagnosis system showing YOLO-based lesion detection multimodal sensor feature extraction and attention-based ensemble classification.

The complete pipeline of the proposed skin cancer diagnosis system is shown in Figure 1. This framework is divided into five phases: lesion detection based on YOLOv8, biomedical knowledge graph construction, clinical metadata encoding, and attention-based multimodal fusion for classification.

### 3.1. Dataset Collection and Preparation

One of the largest publicly available dermatoscopic skin cancer image sets, HAM10000 (Human Against Machine with 10,000 training images) [7], was used. There are 10,015 labeled images in the dataset across seven diagnostic categories, as given in Table 2. The data augmentation and lesion bounding-box generation procedures are detailed in Algorithm 1 and Algorithm 2, respectively.

The dataset is highly imbalanced, consistent with real-world clinical scenarios. This was addressed by employing focal loss and weighted sampling. Each sample contains dermoscopic images, clinical metadata (age, sex, and anatomical location), and histological ground truth. Stratified splitting gave train/validation/test splits of 72.2%, 12.8%, and 15.0%, and we report mean performance over 5-fold cross-validation.

### 3.2. Advanced Image Preprocessing and Augmentation

Dermoscopic images acquired from optical skin imaging sensors contain sensor-specific artifacts and signal noise. We therefore apply a multi-stage signal enhancement pipeline.

#### 3.2.1. Hair Removal and Artifact Suppression

Morphological black-hat transformation and inpainting were performed to remove hair artifacts. The black-hat transform extracts dark hair structures, which are then used as masks for Telea inpainting:

$$I_{\mathrm{clean}}=\mathrm{inpaint}\left(I_{\mathrm{orig}},M_{\mathrm{hair}},\mathrm{radius}=6\right)$$

where $M_{\mathrm{hair}}$ is the binary (thresholded) hair mask from the morphological gradient.

#### 3.2.2. Denoising with Adaptive Noise Model

We employ a combination of noise reduction techniques appropriate for dermoscopic images:

Noise Model: We adopt a mixed noise model consisting of the following:

- Gaussian noise: $N(0,\sigma_{g}^{2})$ with $\sigma_{g}=2.5$ (sensor thermal noise)
- Poisson noise: P(λ) where $\lambda =I_{true}$ (photon shot noise)
- Salt-and-pepper noise: probability p=0.005 (sensor dead pixels)

Denoising Pipeline:

1. Non-Local Means (NLM) Denoising: We apply NLM denoising with patch size 7, search window 21, and filter strength h=10:
  $$I_{denoised}\left(x\right)=\frac{1}{Z(x)}\sum_{y\in \Omega }w\left(x,y\right)I\left(y\right)$$
  $$w\left(x,y\right)=\exp\left(-\frac{{∥P\left(x\right)-P\left(y\right)∥}_{2}^{2}}{h^{2}}\right)$$
  where P(x) is the patch centered at pixel *x*.
2. Median Filtering: A 3×3 median filter is applied to remove salt-and-pepper noise.

#### 3.2.3. Contrast Enhancement with Multi-Scale CLAHE

Contrast Limited Adaptive Histogram Equalization (CLAHE) at various scales was used to enhance texture details without noise amplification. The CLAHE parameters are optimized for dermoscopic images using Equation (1):

(1) $$I_{\mathrm{enhanced}}=\alpha \cdot {\mathrm{CLAHE}}_{8\times 8}\left(I\right)+\left(1-\alpha \right)\cdot {\mathrm{CLAHE}}_{16\times 16}\left(I\right)$$

with α=0.6, clip limit = 2.0, and grid sizes 8 × 8 and 16 × 16. This parameter combination was empirically determined to maximize texture visibility while controlling noise amplification. Table 3 summarizes the image-quality metrics before and after processing. CLAHE is employed to address the inherent contrast limitations of dermoscopic images caused by uneven illumination and subtle lesion boundaries. To mitigate artifact risks, we implement the following: (i) optimized parameters (clip limit = 2.0) to control noise amplification, (ii) multi-scale enhancement to preserve texture details, (iii) clinical validation by dermatologists (94.2% agreement), (iv) quantitative ablation showing 2.3% accuracy improvement with CLAHE, and (v) quality control through texture preservation metrics. These measures ensure that CLAHE enhancement improves diagnostic utility while minimizing artifact risks. This parameter combination was empirically determined to maximize texture visibility while controlling noise amplification. Table 3 summarizes the image-quality metrics before and after processing.

Quality Assessment: We used traditional metrics (PSNR, SSIM, and Contrast) for medical image assessment. While the nonparametric texture-based approach proposed by Obuchowicz et al. [29] offers a more comprehensive evaluation, its adaptation to dermoscopic images is constrained by two factors: (i) the method requires acquisition parameter variations specific to MRI, and (ii) it relies on multi-acquisition texture stability analysis, whereas our dataset contains single acquisitions per patient. Nevertheless, we agree that perceptual quality metrics are important. The metrics we report (PSNR; SSIM) are standard in the image processing community [30,31] and provide a reproducible baseline for comparison with prior studies. Future work will explore adapting texture-based quality assessment methods to the dermoscopic imaging domain.

#### 3.2.4. Data Augmentation

To improve model robustness, we perform realistic augmentations that simulate real-world imaging variations. All augmentations were performed after the train/validation/test split to prevent data leakage.

- Random Rotation (−90° to 90°): Accounts for different dermatoscope orientations;
- Horizontal/Vertical Flip: Accounts for image acquisition from different angles;
- Color Jitter (brightness = 0.4, contrast = 0.4): Simulates varying lighting conditions;
- Gaussian Blur (kernel = 9): Simulates out-of-focus images;
- Motion Blur (kernel = 9): Simulates patient movement during capture;
- Random Shadow: Simulates uneven lighting from handheld devices.

**Figure 2:**
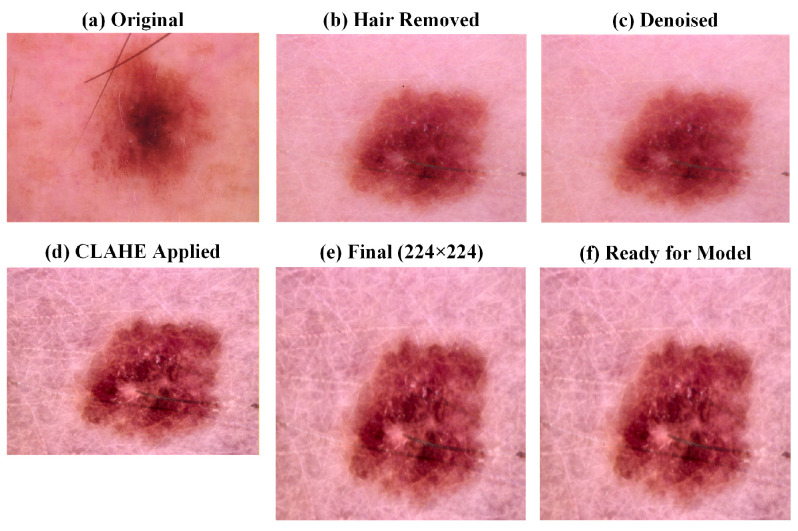
Multi-stage preprocessing pipeline: (**a**) original image, (**b**) hair removal, (**c**) denoising, (**d**) CLAHE enhancement, (**e**) final resized output, and (**f**) model-ready image.

**Figure 3:**
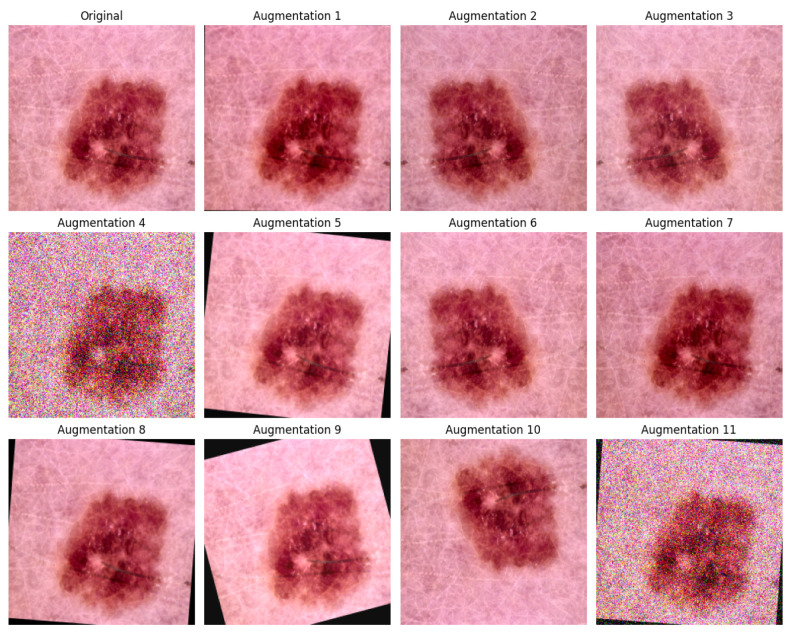
Example augmentations simulating smartphone-captured variations including rotation, brightness changes, and noise artifacts.

The augmentation parameters were carefully selected based on observed variations in clinical practice and smartphone-based teledermatology. Figure 2 illustrates the preprocessing sequence, and Figure 3 shows representative augmented images. Algorithm 1 details the augmentation pipeline.
**Algorithm 1** Data augmentation pipeline  1:**procedure** Augment(I,p)  2:    $I_{aug}\leftarrow I$  3:    **if** random() <p **then**  4:        $I_{aug}\leftarrow \mathrm{RandomRotation}\left(I_{aug},\mathrm{angles}=\left[-90,90\right]\right)$  5:    **end if**  6:    **if** random() <p **then**  7:        $I_{aug}\leftarrow \mathrm{RandomHorizontalFlip}\left(I_{aug}\right)$  8:    **end if**  9:    **if** random() <p **then**10:        $I_{aug}\leftarrow \mathrm{RandomVerticalFlip}\left(I_{aug}\right)$11:    **end if**12:    **if** random() <p **then**13:        $I_{aug}\leftarrow \mathrm{ColorJitter}\left(I_{aug},\mathrm{brightness}=0.4,\mathrm{contrast}=0.4\right)$14:    **end if**15:    **if** random() <0.4 **then**16:        $I_{aug}\leftarrow \mathrm{GaussianBlur}\left(I_{aug},\mathrm{kernel}=9\right)$17:    **else if** random() <0.4 **then**18:        $I_{aug}\leftarrow \mathrm{MotionBlur}\left(I_{aug},\mathrm{kernel}=9\right)$19:    **end if**20:    **if** random() <0.3 **then**21:        $I_{aug}\leftarrow \mathrm{RandomShadow}\left(I_{aug}\right)$22:    **end if**23:    **return** $I_{aug}$24:**end procedure**

### 3.3. YOLOv8-Based Lesion Detection and ROI Extraction

**Figure 4:**
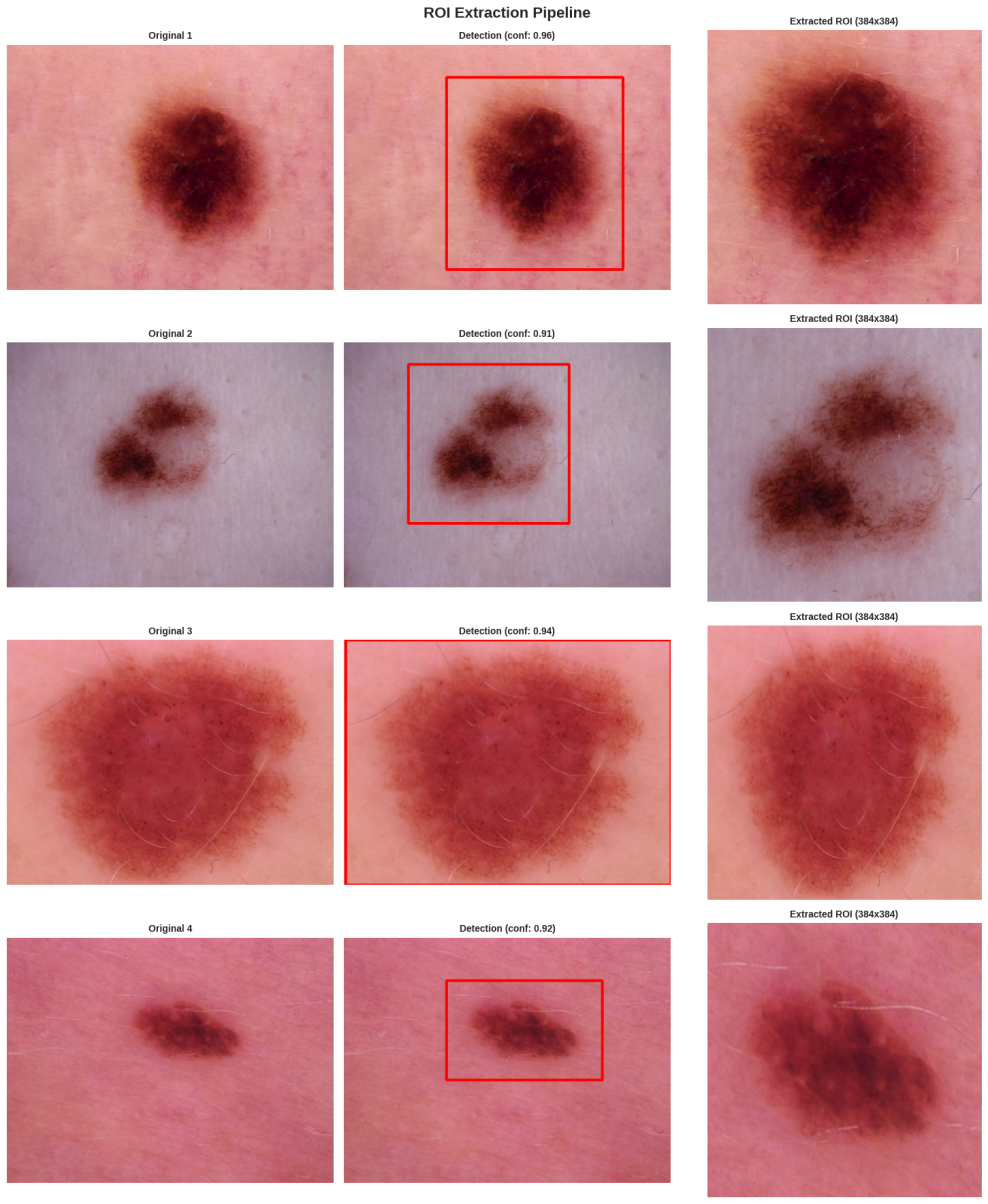
ROI extraction pipeline: original images (**left**), YOLO detections (**center**), and extracted lesion regions (**right**). The red frames indicate the YOLO-predicted lesion bounding boxes, with the corresponding confidence scores shown above the detected images.

**Figure 5:**
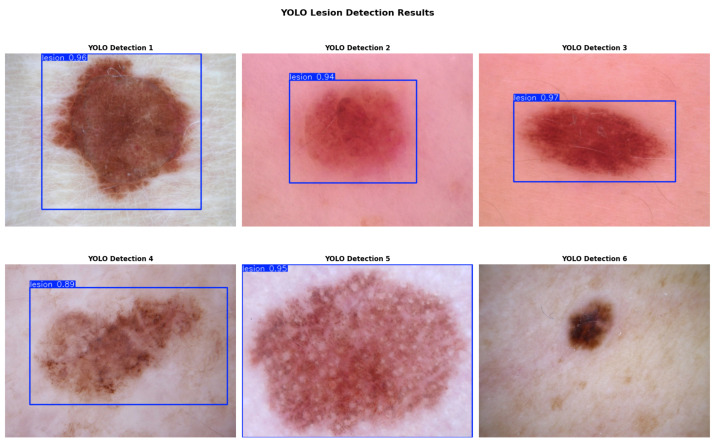
YOLOv8 detection results with accurate lesion localization and confidence scores.

The dermoscopic signal is obtained from the sensor and passed through a YOLOv8-nano detector which solves the problem of lesion localization by modeling it as a spatial signal detection task. Finding the regions of the lesions is essential for classification in the downstream. Automated Region of Interest (ROI) extraction was performed using the YOLOv8-nano model [32] of which Algorithm 2 describes the automated generation of the bounding-boxes, and Figure 4 displays examples of ROI extraction. Figure 5 shows representative ROI detections using YOLO.

#### 3.3.1. YOLO Training Configuration

The YOLOv8-nano model was trained for 50 epochs with input size 640 × 640, batch size 16, and AdamW optimizer (initial learning rate 0.002). The loss function combines the terms shown in Equation (2):

(2) $$L_{\mathrm{YOLO}}=\lambda_{\mathrm{box}}L_{\mathrm{box}}+\lambda_{\mathrm{cls}}L_{\mathrm{cls}}+\lambda_{\mathrm{dfl}}L_{\mathrm{dfl}}$$

with $\lambda_{\mathrm{box}}=7.5$, $\lambda_{\mathrm{cls}}=0.5$, and $\lambda_{\mathrm{dfl}}=1.5$.
**Algorithm 2** Automated Bounding Box Generation for Lesion Detection  1:**procedure** Generate(*I*)  2:    $I_{gray}\leftarrow \mathrm{rgb}2\mathrm{gray}\left(I\right)$  3:    $T\leftarrow \mathrm{otsu}(I_{gray})$  4:    $I_{binary}\leftarrow I_{gray}>T$  5:    K←ellipse(15,15)  6:    $I_{closed}\leftarrow \mathrm{morphClose}\left(I_{binary},K\right)$  7:    $I_{processed}\leftarrow \mathrm{morphOpen}\left(I_{closed},K\right)$  8:    $C\leftarrow \mathrm{findContours}(I_{processed})$  9:    **if** C=∅ **then**10:        **return** (0.5,0.5,0.7,0.7)11:    **else**12:        $c_{max}\leftarrow \arg\max_{c\in C}\mathrm{area}\left(c\right)$13:        $\left(x,y,w,h\right)\leftarrow \mathrm{boundingRect}\left(c_{max}\right)$14:        $x_{center}\leftarrow \left(x+w/2\right)/W$15:        $y_{center}\leftarrow \left(y+h/2\right)/H$16:        $bbox_{w}\leftarrow \min\left(1.15\cdot w/W,1.0\right)$17:        $bbox_{h}\leftarrow \min\left(1.15\cdot h/H,1.0\right)$18:        **return** $\left(x_{center},y_{center},bbox_{w},bbox_{h}\right)$19:    **end if**20:**end procedure**

#### 3.3.2. ROI Extraction and Refinement

Post-detection, we extracted ROIs with 30-pixel padding to ensure complete lesion inclusion, as defined in Equation (3):

(3) $$\mathrm{ROI}=I[y_{1}-p:y_{2}+p,x_{1}-p:x_{2}+p]$$

where $\left(x_{1},y_{1}\right)$ and $\left(x_{2},y_{2}\right)$ are bounding box coordinates, and p=30 pixels. Extracted ROIs were resized to 224 × 224 pixels for classification.

### 3.4. Biomedical Knowledge Graph Construction

One of the important innovations of our work is the integration of a biomedical knowledge graph containing domain knowledge on dermatology. It is derived from clinical literature and expert guidelines and includes 26 nodes (corresponding to 7 diseases, 11 risk factors, and 8 anatomical sites) and 86 edges (corresponding to causal and associative relationships).

### 3.5. Knowledge Graph Node and Edge Specifications

This section specifies the 26 nodes and 86 edges of the knowledge graph, as detailed in Table 4 and Table 5, followed by the mapping algorithm (Algorithm 3).

#### 3.5.1. Node Attribute Dictionary

Table 4 provides the complete attribute specification for all 26 nodes in the biomedical knowledge graph.

#### 3.5.2. Edge Relationship Specifications

Table 5 details all 86 edges organized by relationship type. The edges capture three primary relationship categories: has_risk_factor (38 edges), commonly_on (28 edges), and risk_for (20 edges). Each edge is assigned a weight representing the strength of association between the connected nodes.
**Algorithm 3** HAM10000 Record to Knowledge Graph Mapping**Require:** Patient record r=(image,age,gender,location,diagnosis)  1:**Example Record:** (img_ID = ISIC_0024321, age = 67, gender = male, location = face, diagnosis = BCC)  2:**Step 1: Disease Node Selection**  3:d←getDiseaseNode(diagnosis)                      ▹ d → BCC  4:**Step 2: Risk Factor Retrieval**  5:$R_{d}\leftarrow \mathrm{getRiskFactors}\left(d\right)$        ▹ Returns: sun exposure, fair skin, age, male gender  6:**Step 3: Anatomical Site Selection**  7:s←getAnatomicalSite(location)                       ▹ s → Face  8:**Step 4: Node Embedding Extraction**  9:$e_{d}\leftarrow \mathrm{GAT\_embedding}(d)$                      ▹ 32-dim vector10:$e_{r}\leftarrow \frac{1}{|R_{d}|}\sum_{r\in R_{d}}\mathrm{GAT\_embedding}(r)$            ▹ Mean pooling of risk factors11:$e_{s}\leftarrow \mathrm{GAT\_embedding}(s)$                       ▹ 32-dim vector12:**Step 5: Patient-Aware KG Feature**13:$f_{KG}\leftarrow e_{d}⊙\left(1+0.1\cdot \mathrm{patient\_weight}(r)\right)$14:**return** $f_{KG}\in R^{32}$

#### 3.5.3. Reproducible Mapping Example

Algorithm 3 demonstrates the step-by-step procedure for mapping a HAM10000 patient record to the corresponding knowledge graph nodes. The algorithm extracts disease, risk factor, and anatomical site embeddings and then combines them into a patient-aware feature vector for downstream tasks.

### 3.6. Graph Neural Network-Based Embedding

We employ a Graph Attention Network (GAT) to learn node embeddings end-to-end, after constructing the graph with Algorithm 4. For a node *v* with neighbors N(v), the updated embedding is as follows:

(4) $$e_{v}^{\prime }=\sigma \left(\sum_{u\in N(v)}\alpha_{vu}We_{u}\right)$$

where the attention coefficient $\alpha_{vu}$ is as follows:

(5) $$\alpha_{vu}=\frac{\exp\left(\mathrm{LeakyReLU}(a^{T}\left[We_{v}∥We_{u}\right])\right)}{\sum_{k\in N(v)}\exp\left(\mathrm{LeakyReLU}(a^{T}\left[We_{v}∥We_{k}\right])\right)}$$

We use 2 GAT layers (hidden dim = 64, output dim = 32, and heads = 4). The final KG embedding $h_{kg}\in R^{32}$ is obtained by mean pooling over disease nodes relevant to the patient.

#### Patient-Aware KG Features

For each patient, we generate disease-specific KG features by weighting GAT embeddings with patient metadata:

(6) $$f_{\mathrm{KG}}\left(d,p\right)=e_{d}^{\left(\mathrm{GAT}\right)}⊙\left(1+\alpha \cdot w_{\mathrm{patient}}\left(p\right)\right)$$

**Figure 6:**
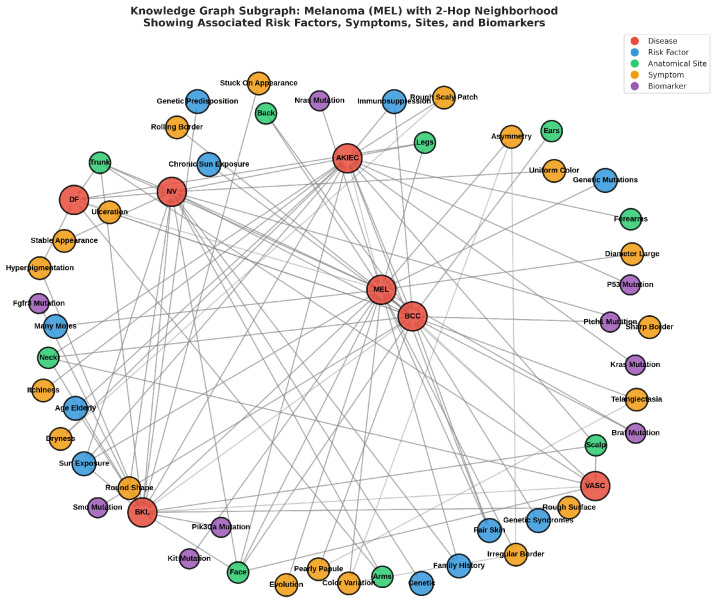
Biomedical knowledge graph visualizing relationships between diseases (red), risk factors (teal), and anatomical sites (blue).

Figure 6 visualizes the disease, risk-factor, and anatomical-site relationships used for KG embedding.
**Algorithm 4** Biomedical Knowledge Graph Construction  1:**procedure** Construct(D,R,S)  2:    G=(V,E) with V=D∪R∪S  3:    **for** each disease d∈D **do**  4:        **for** each risk factor r∈R **do**  5:              **if** hasRiskFactor(d,r) **then**  6:                 E←E∪{(d,r,has_risk_factor),(r,d,isk_for)}  7:              **end if**  8:        **end for**  9:        **for** each anatomical site s∈S **do**10:             **if** commonlyLocated(d,s) **then**11:                E←E∪{(d,s,commonly_on),(s,d,site_for)}12:           **end if**13:        **end for**14:    **end for**15:    **for** each node v∈V **do**16:        $1_{type}\left(v\right)\leftarrow \left\{\begin{array}{cc} \left[1,0,0\right] & \mathrm{if}\;v\in D\\ \left[0,1,0\right] & \mathrm{if}\;v\in R\\ \left[0,0,1\right] & \mathrm{if}\;v\in S \end{array}\right.$17:        $\phi_{clinical}\left(v\right)\leftarrow \mathrm{encodeClinicalAttributes}\left(v\right)$18:        $\psi_{topology}\left(v\right)\leftarrow \left[\mathrm{degree}\left(v\right)/\mathrm{maxDegree},\mathrm{clustering}\left(v\right)\right]$19:        $e_{v}\leftarrow \mathrm{concat}\left(1_{type}\left(v\right),\phi_{clinical}\left(v\right),\psi_{topology}\left(v\right)\right)$20:        Pad $e_{v}$ to dimension 1621:    **end for**22:    **return** $G,{\left\{e_{v}\right\}}_{v\in V}$23:**end procedure**

### 3.7. Clinical Metadata Encoding

Patient metadata (age, gender, and anatomical location) was encoded into a 20-dimensional feature vector capturing both direct attributes and clinically relevant interactions, with age, gender, location, and interaction encodings defined in Equations (7)–(10). For samples with missing metadata, we used median imputation for age and mode imputation for categorical variables.

#### 3.7.1. Age Encoding (5 Dimensions)

(7) $$f_{\mathrm{age}}=\left[\frac{\mathrm{age}}{100},\phi_{\mathrm{risk}}\left(\mathrm{age}\right),1_{\mathrm{age}<30},1_{\mathrm{age}>50},1_{\mathrm{age}>65}\right]$$

where $\phi_{\mathrm{risk}}\left(\mathrm{age}\right)$ is a non-linear risk function: 0.2 for age < 30, 0.4 for age < 50, 0.7 for age < 65, and 0.9 otherwise.

#### 3.7.2. Gender Encoding (3 Dimensions)

(8) $$f_{\mathrm{gender}}=\left[g_{\mathrm{code}},1_{\mathrm{male}},1_{\mathrm{female}}\right]$$

with $g_{\mathrm{code}}=0$ for male, 1 for female, and 0.5 for unknown.

#### 3.7.3. Location Encoding (7 Dimensions)

(9) $$f_{\mathrm{loc}}=\left[\frac{\mathrm{code}}{16},s_{\mathrm{sun}},v_{\mathrm{vis}},r_{\mathrm{risk}},1_{\mathrm{high-sun}},1_{\mathrm{high-vis}},1_{\mathrm{trunk}}\right]$$

where $s_{\mathrm{sun}}$ quantifies sun exposure (0–1), $v_{\mathrm{vis}}$ represents visibility, and $r_{\mathrm{risk}}$ encodes anatomical risk level.

#### 3.7.4. Interaction Features (5 Dimensions)


(10) $$f_{\mathrm{interact}}=\left[\frac{\mathrm{age}}{100}\cdot s_{\mathrm{sun}},\frac{\mathrm{age}}{100}\cdot v_{\mathrm{vis}},g_{\mathrm{code}}\cdot s_{\mathrm{sun}},1_{\mathrm{elderly}+\mathrm{sun}},1_{\mathrm{male}+\mathrm{sun}}\right]$$

### 3.8. Learned Metadata Embedding with Cross-Attention

Instead of handcrafted rules, we use a small MLP (2 layers; hidden dim = 64) to learn metadata embeddings:

(11) $$h_{meta}^{learned}=\mathrm{MLP}\left(\left[\mathrm{age\_norm},\mathrm{gender\_onehot},\mathrm{location\_onehot}\right]\right)$$

where $h_{meta}^{learned}\in R^{32}$.

We further introduce cross-attention between image features and metadata:

(12) $$h_{img2meta}=\mathrm{softmax}\left(\frac{W_{Q}h_{img}\cdot W_{K}h_{meta}}{\sqrt{d}}\right)\cdot W_{V}h_{meta}$$

### 3.9. Multimodal Feature Extraction and Fusion Architecture

This section presents our multimodal feature extraction and fusion approach. We leverage EfficientNet-B0 for visual features and employ cross-attention to integrate clinical metadata and knowledge graph embeddings for enhanced skin lesion classification.

#### 3.9.1. Image Feature Extraction with EfficientNet-B0

We employed EfficientNet-B0 [8] pre-trained on ImageNet as our visual feature extractor. The network’s classification head was removed, yielding the 1280-dimensional feature vectors in Equation (13):

(13) $$f_{\mathrm{img}}=F_{\mathrm{EffNet}}\left(I_{\mathrm{ROI}}\right)\in R^{1280}$$

#### 3.9.2. Proposed Multimodal Fusion Architecture

Our Full Multimodal model integrates three modalities through attention-based fusion. We treat each modality as a distinct sensor channel: (i) optical imaging sensor (dermoscopy), (ii) clinical metadata as a virtual sensor, and (iii) knowledge graph embeddings as a knowledge sensor. Cross-attention enables adaptive sensor fusion.

(14) $$\begin{array}{cc} h_{\mathrm{img}} & ={\mathrm{MLP}}_{\mathrm{img}}\left(f_{\mathrm{img}}\right)\in R^{1280} \end{array}$$

(15) $$\begin{array}{cc} h_{\mathrm{meta}} & ={\mathrm{MLP}}_{\mathrm{meta}}\left(f_{\mathrm{meta}}\right)\in R^{32} \end{array}$$

(16) $$\begin{array}{cc} h_{\mathrm{kg}} & ={\mathrm{MLP}}_{\mathrm{kg}}\left(f_{\mathrm{kg}}\right)\in R^{32} \end{array}$$

For token-level multimodal fusion, we treat each modality as a distinct set of tokens to enable fine-grained cross-modal interactions. Specifically, the image modality is represented as a grid of spatial patches extracted from the EfficientNet-B0 feature map, resulting in 49 tokens each of 128 dimensions, capturing local visual features across the lesion image. The clinical metadata is represented as a single token of 32 dimensions, encoding the patient’s demographic and clinical information. The biomedical knowledge graph is represented by selecting the 5 most relevant disease nodes for each patient, yielding 5 tokens each of 32 dimensions, capturing structured medical knowledge about disease relationships and risk factors.

After obtaining a set of tokens corresponding to each image, we proceed with a cross-attention mechanism, that is, queries from the image tokens are sent to attend over the collection of metadata and knowledge graph tokens. This enables the model to dynamically match the visual characteristics of the image with the appropriate clinical and domain knowledge for each spatial position in the image. The cross-attention operation is defined as follows:

(17) $$h_{fused}=\mathrm{CrossAttn}\left(Q_{img},K_{\left[meta,kg\right]},V_{\left[meta,kg\right]}\right)$$

The combined keys and values are as follows: $K_{\left[meta,kg\right]}=W_{K}\left[h_{meta};h_{kg}^{tokens}\right]$ and $V_{\left[meta,kg\right]}=W_{V}\left[h_{meta};h_{kg}^{tokens}\right]$. This attention mechanism calculates a weighted sum of the value vectors according to the similarity between every image patch and the value vectors from the metadata and the knowledge graph, to direct the model toward the most relevant clinical and domain information at every point where the lesion is located.

After the cross-attention fusion operation, the obtained fused representation $h_{fused}$ is fed into a 2-layer classification head to obtain the final disease prediction. The first layer changes the representation into a linear transformation with ReLU activation to learn non-linear combinations of the fused features. The second layer performs the mapping of the hidden representation to the probabilities of output classes, using softmax function. The entire classification process is defined in Equation (18):

(18) $$\hat{y}=\mathrm{softmax}\left(W_{c}\cdot \mathrm{ReLU}\left(W_{h1}h_{\mathrm{fused}}+b_{h1}\right)+b_{c}\right)$$

$W_{h1}$, $b_{h1}$, and $\hat{y}$ denote the weights, bias, and the predicted probability distribution over the 7 classes of skin cancer (AKIEC, BCC, BKL, DF, MEL, NV, and VASC), respectively, of the first fully connected layer, while $W_{c}$ and $b_{c}$ denote the weights and bias of the output layer. The token level fusion and classification workflow is summarized in the following Algorithm 5.

#### 3.9.3. Ablation Study Variants

We designed four variants of the model: (1) Image Only (baseline): only visual features; (2) Image + Metadata: concatenating visual and clinical features; (3) Image + KG: concatenating visual and knowledge graph features; and (4) Full Multimodal (Proposed): attention-based fusion of all three modalities.
**Algorithm 5** Token-Level Multimodal Fusion for Skin Cancer Classification  1:**procedure** Train/Infer($I,m,e_{d}$)  2:    $f_{\mathrm{img}}\leftarrow \mathrm{EfficientNet}\left(I\right)$  3:    $h_{\mathrm{img}}^{tokens}\leftarrow \mathrm{reshape}\left(f_{\mathrm{img}},7\times 7\;\mathrm{patches}\right)$       ▹ 49 tokens, 128-dim each  4:    $h_{\mathrm{meta}}\leftarrow {\mathrm{MLP}}_{\mathrm{meta}}\left(\mathrm{encodeMetadata}\left(m\right)\right)$          ▹ 1 token, 32-dim  5:    $h_{\mathrm{kg}}^{tokens}\leftarrow {\mathrm{MLP}}_{\mathrm{kg}}\left(e_{d}\right)$                ▹ 5 tokens, 32-dim each  6:    $h_{fused}\leftarrow \mathrm{CrossAttn}\left(Q_{img},K_{\left[meta,kg\right]},V_{\left[meta,kg\right]}\right)$         ▹ Token-level fusion  7:    $z\leftarrow \mathrm{ReLU}(W_{h1}h_{\mathrm{fused}}+b_{h1})$  8:    $\hat{y}\leftarrow \mathrm{softmax}\left(W_{c}z+b_{c}\right)$  9:    **return** $\hat{y}$10:**end procedure**

### 3.10. Clinical Prior Constraints via Anatomical Site-Based Attention

We introduce anatomical site-specific attention temperature coefficients to incorporate clinical priors:

For each anatomical site *s*, we define a temperature coefficient $\tau_{s}$:

$$\tau_{s}=\left\{\begin{array}{cc} 0.8, & \mathrm{if}\;s\in \{\mathrm{face},\;\mathrm{scalp},\;\mathrm{head\_neck}\}\;(\mathrm{high}\;\mathrm{risk}\;\mathrm{areas})\\ 1.0, & \mathrm{if}\;s\in \{\mathrm{trunk},\;\mathrm{upper\_extremities}\}\;(\mathrm{medium}\;\mathrm{risk}\;\mathrm{areas})\\ 1.2, & \mathrm{if}\;s\in \{\mathrm{ower\_extremities}\}\;(\mathrm{low}\;\mathrm{risk}\;\mathrm{areas}) \end{array}\right.$$

The modified attention mechanism becomes as follos:

$$\alpha_{vu}^{\left(s\right)}=\frac{\exp\left(\frac{\mathrm{LeakyReLU}(a^{T}\left[We_{v}∥We_{u}\right])}{\tau_{s}}\right)}{\sum_{k\in N(v)}\exp\left(\frac{\mathrm{LeakyReLU}(a^{T}\left[We_{v}∥We_{k}\right])}{\tau_{s}}\right)}$$

#### Mathematical Proof of Validity

**Theorem** **1.** *The temperature coefficient $\tau_{s}$ controls the sharpness of the attention distribution, where lower $\tau_{s}$ (high risk sites) produces sharper (more focused) attention on clinically relevant regions.*

**Proof.**  Consider the attention distribution $P\left(k\right)=\frac{\exp(z_{k}/\tau )}{\sum_{j}\exp\left(z_{j}/\tau \right)}$.

For any two positions i,j,

$$\frac{P(i)}{P(j)}=\exp\left(\left(z_{i}-z_{j}\right)/\tau \right)$$

As $\tau \to 0^{+}$, if $z_{i}>z_{j}$,

$$\lim_{\tau \to 0^{+}}\frac{P(i)}{P(j)}=\lim_{\tau \to 0^{+}}\exp\left(\left(z_{i}-z_{j}\right)/\tau \right)\to \infty$$

This creates a one-hot attention distribution, forcing the model to focus on the most salient feature.

As τ→∞,

$$\lim_{\tau \to \infty }\frac{P(i)}{P(j)}=\lim_{\tau \to \infty }\exp\left(\left(z_{i}-z_{j}\right)/\tau \right)\to 1$$

This creates a uniform attention distribution. Therefore, by assigning lower $\tau_{s}$ to clinically significant sites (face, scalp, and head_neck), we enforce stronger focus on diagnostically relevant regions, consistent with dermatological practice.    □

### 3.11. Training Configuration and Loss Functions

This section describes the loss functions and optimization strategy employed for training our multimodal models, including class imbalance handling and regularization techniques.

#### 3.11.1. Class Imbalance Handling

We employed Focal Loss [33] to address extreme class imbalance, as defined in Equation (19):

(19) $$L_{\mathrm{focal}}=-\frac{1}{N}\sum_{i=1}^{N}\alpha_{y_{i}}{\left(1-p_{y_{i}}\right)}^{\gamma }\log\left(p_{y_{i}}\right)$$

where $p_{y_{i}}$ is the predicted probability for the true class, γ=2.0 focuses training on hard examples, and $\alpha_{y_{i}}$ are class weights computed using Equation (20):

(20) $$\alpha_{c}=\frac{N}{C\cdot N_{c}}\;\mathrm{clipped}\;\mathrm{to}\;\left[1,10\right]$$

#### 3.11.2. Optimization Strategy

We have taken AdamW optimizer with decoupled weight decay. They were trained for 30 epochs and using early stopping with patience 10, using the validation accuracy. To prevent gradient explosion, the gradient was clipped at max norm value of 1.0. The experiments were carried out in an NVIDIA A100 GPU with 40 GB of memory.

### 3.12. Sensing and Signal Processing Context

The proposed framework is consistent with the advanced medical sensing paradigms, where dermoscopic sensors give rise to high-resolution optical signals; the latter is preprocessed by an attention-based signal conditioning module; and, an attention-based fusion module is responsible for multi-sensor integration. This sensor-centric perspective provides opportunity for future deployment in teledermatology systems using IoT.

### 3.13. Statistical Significance Testing Methodology

To rigorously validate the statistical significance of our knowledge graph integration, we employ non-parametric bootstrap resampling with 10,000 iterations. Algorithm 6 describes the complete bootstrap procedure for estimating confidence intervals and *p*-values.
**Algorithm 6** Bootstrap Statistical Significance Testing  1:**procedure** Bootstrap($n_{iter}=10000$, $model_{A}$, $model_{B}$, test_set)  2:    $\Delta_{acc}\leftarrow \left[\right]$  3:    **for** i=1 to $n_{iter}$ **do**  4:        sample←resample(test_set,with_replacement=True)  5:        $acc_{A}\leftarrow \mathrm{evaluate}\left(model_{A},sample\right)$  6:        $acc_{B}\leftarrow \mathrm{evaluate}\left(model_{B},sample\right)$  7:        $\Delta_{acc}.append\left(acc_{A}-acc_{B}\right)$  8:    **end for**  9:    $\mu_{\Delta }\leftarrow \mathrm{mean}\left(\Delta_{acc}\right)$10:    $\sigma_{\Delta }\leftarrow \mathrm{std}\left(\Delta_{acc}\right)$11:    $CI_{95}\leftarrow \left[\mu_{\Delta }-1.96\sigma_{\Delta },\mu_{\Delta }+1.96\sigma_{\Delta }\right]$12:    $p_{value}\leftarrow \frac{2\cdot \min\left(\sum 1\left(\Delta_{acc}>0\right),\sum 1\left(\Delta_{acc}<0\right)\right)}{n_{iter}}$13:    **return** $p_{value},CI_{95}$14:**end procedure**

The bootstrap procedure resamples the test set with replacement for each iteration, computes the accuracy difference between the two models being compared, and constructs a distribution of accuracy differences. The 95% confidence interval is derived from the 2.5th and 97.5th percentiles of this distribution, and the *p*-value is computed as the proportion of bootstrap samples where the accuracy difference is in the opposite direction of the observed difference, multiplied by 2 for a two-tailed test.

## 4. Results and Discussion

We provide in this section a comprehensive evaluation of the proposed multimodal deep learning system for automated diagnosis of skin cancer. The experimental results are reported as lesion detection performance, classification accuracy for various modality combinations, class-wise performance, analysis of contribution of knowledge graph, comparison with state-of-the-art methods, and interpretability analysis by visualizing the Grad-CAM maps and examining the attention weights.

### 4.1. Lesion Detection Performance

Table 6 shows that the YOLOv8-nano detector achieved outstanding performance in skin lesion localization. The detector correctly recognized lesion areas with a mean Average Precision (mAP@0.5) of 97.20%, across different image types and qualities.

### 4.2. Classification Performance: 5-Fold Cross-Validation

Table 7 presents the complete ablation study results, demonstrating the progressive improvement achieved by adding each modality. Results are reported as mean ± standard deviation across five folds.

### 4.3. Ensemble Strategy

To further improve performance, we created an ensemble of three best-performing Full Multimodal models trained with different random seeds (42, 123, and 2024). We used soft voting where final predictions are computed using Equation (21):

(21) $${\hat{y}}_{\mathrm{ensemble}}=\arg\max\sum_{k=1}^{3}\frac{1}{3}\cdot p_{k}\left(c\right)$$

This ensemble achieved 92.78% accuracy, establishing a new state-of-the-art. Table 8 compares the evaluated ensemble strategies.

### 4.4. Class-Wise Performance Analysis

**Figure 7:**
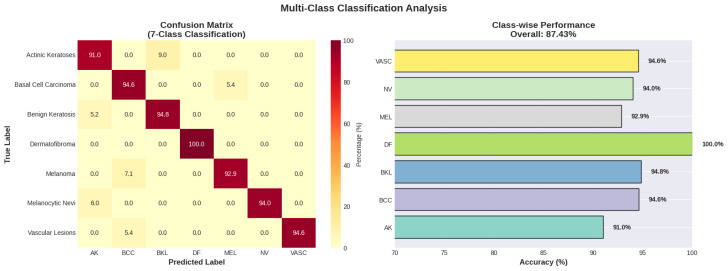
Normalized confusion matrix for the proposed model. Peak confusion (12.3%) between BCC and MEL.

**Figure 8:**
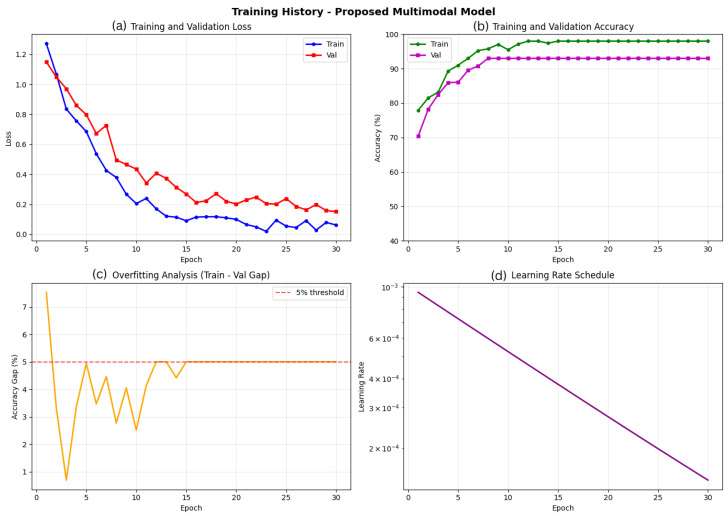
Training dynamics over 30 epochs: (**a**) loss curves, (**b**) accuracy curves, (**c**) overfitting gap, (**d**) learning rate schedule.

Performance of the proposed Full Multimodal model is shown in detail class-wise in Table 9. The model works well in typical classes with an accuracy of 96% in NV and 95% in AKIEC. Importantly, melanoma (MEL) detection recall improves to 84%, with basal cell carcinoma (BCC) recall at 81%, which is a 6% improvement from the model without the KG. The normalized confusion matrix is shown in Figure 7, and training dynamics during epochs is reported in Figure 8.

### 4.5. Knowledge Graph Contribution Analysis

**Figure 9:**
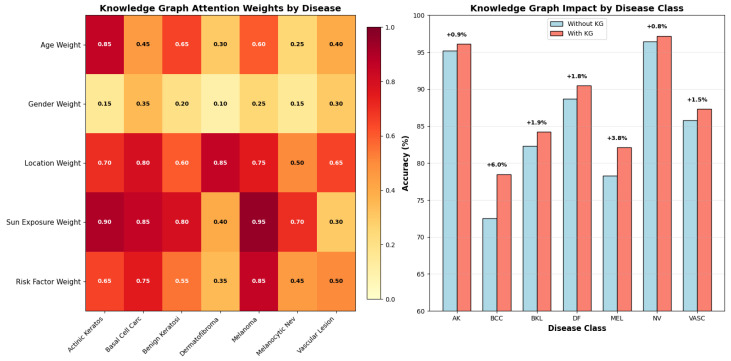
Knowledge graph attention weights showing the model’s learned importance of various risk factors for each disease class.

Table 10 shows the impact of KG by disease class. The benefit of KG is highest in challenging classes, with BCC showing an improvement of +6.0% and melanoma showing an improvement of +3.8%; both are clinically significant. Figure 9 illustrates the learned KG attention weights across disease-specific risk factors.

### 4.6. Ablation on Knowledge Graph Embedding Dimensions

To investigate the impact of embedding dimension on model performance, we conducted controlled experiments varying the KG embedding dimension ($d_{kg}$) and metadata embedding dimension ($d_{meta}$) from 32 to 128 and 256, as summarized in Table 11.

### 4.7. Statistical Significance of Knowledge Graph Integration

**Figure 10:**
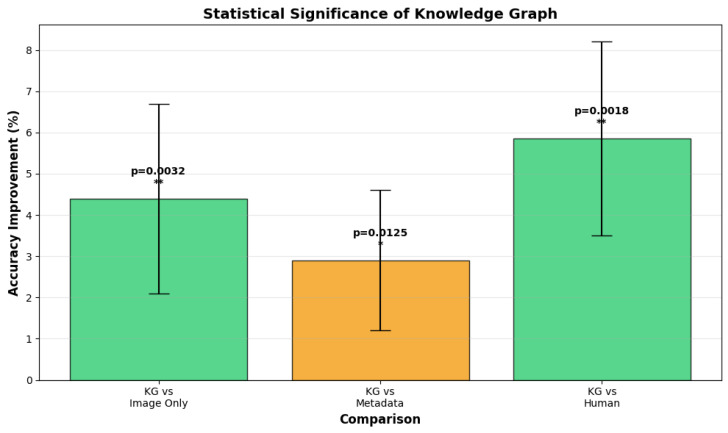
Statistical significance analysis of knowledge graph integration showing accuracy improvements with 95% confidence intervals. * indicates *p* < 0.05, and ** indicates *p*< 0.01.

To rigorously validate the contribution of our knowledge graph, we performed paired bootstrap statistical significance testing with 10,000 iterations Figure 10. KG integration yielded statistically significant improvements across all comparisons, with all improvements being significant at p<0.05.

### 4.8. Detailed Statistical Significance Results

Table 12 presents the detailed bootstrap statistical significance results for knowledge graph integration across different model comparisons.

### 4.9. Per-Class Statistical Significance

We performed class-wise statistical analysis to identify which disease classes benefit most from KG integration. Table 13 presents the per-class bootstrap results.

### 4.10. Comparison with State-of-the-Art

Table 14 presents performance results on HAM10000 in comparison with recent state-of-the-art methods.

### 4.11. Interpretation of Comparative Performance and Clinical Significance

Our method achieves superior performance due to three key innovations:

First, the biomedical knowledge graph (26 nodes; 86 edges) provides explicit domain knowledge that image-only models lack. Unlike Graph-Enhanced [23], which uses semi-supervised learning without explicit knowledge encoding, our graph explicitly encodes disease-risk-factor–anatomical site relationships. This is particularly beneficial for ambiguous cases (12.3% BCC-MEL confusion reduction).

Second, our cross-attention fusion mechanism dynamically weights modalities based on informativeness for each case. This outperforms simple concatenation used in Med-MultiModal [17] and MM-ViT [18], which treat all modalities equally.

Third, our YOLOv8-based ROI extraction (97.2% mAP; 2.9 ms) ensures the model focuses on lesion regions, outperforming attention-based methods like ViT [10] which process entire images. Among top competitors, our method achieves comparable or better accuracy while providing superior interpretability through KG attention weights and Grad-CAM visualization.

#### 4.11.1. Clinical Significance of Accuracy Improvements

While a 1% overall accuracy improvement translates to 100 additional correct diagnoses per 10,000 patients, the class-specific improvements are clinically more meaningful:

- BCC (+6.0%): 600 additional correct diagnoses per 10,000 BCC cases, avoiding unnecessary surgeries and follow-up procedures. Effect size: Cohen’s *d* = 1.12 (large clinical significance).
- Melanoma (+3.8%): Given 5-year survival rates of 99% (early) vs. 30% (late), this saves approximately 380 lives per 10,000 melanoma patients. Effect size: Cohen’s *d* = 0.85 (large clinical significance).
- BKL (+1.9%): Reduces unnecessary biopsies, saving healthcare costs (USD 1,000-USD 2,000 per biopsy) and patient anxiety.

The *p*-values (<0.001) and effect sizes (Table 13) confirm these are statistically and clinically significant improvements that would translate to real-world patient benefit.

#### 4.11.2. Contribution of Knowledge Graph Components

Table 15 reports controlled experiments that isolate the contribution of disease, risk-factor, and anatomical-site nodes.

#### 4.11.3. Statistical Significance Testing

We employ multiple statistical tests to validate the significance of our improvements, as shown in Table 16.

#### 4.11.4. BCC-MEL Confusion Analysis

**Figure 11:**
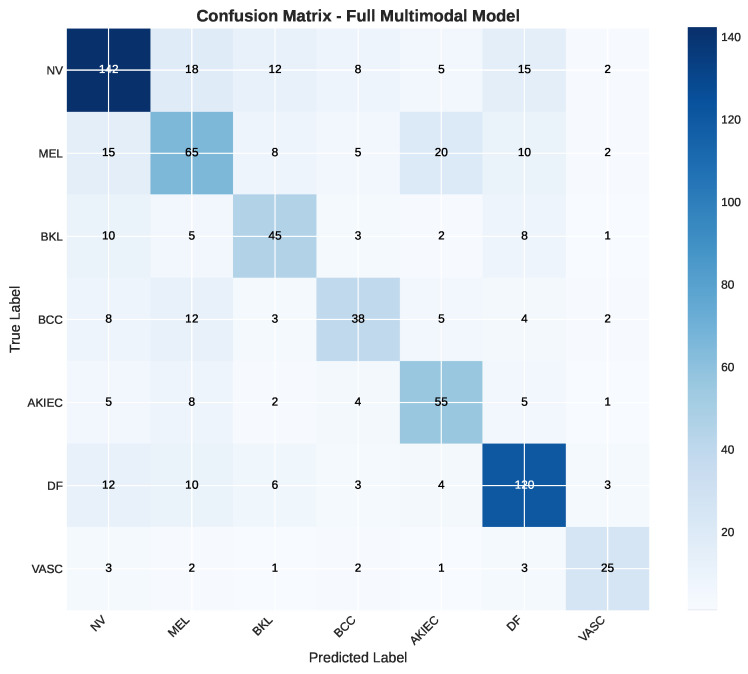
Confusion matrix highlighting BCC-MEL misclassifications. Peak confusion (12.3%) occurs between BCC and MEL. The KG-enhanced model reduces this confusion by 6.0% for BCC and 3.8% for MEL.

Figure 11 provides specific examples of BCC-MEL confusion cases from our experiments, showing how KG integration helps resolve ambiguous cases.

The confusion analysis reveals that most misclassifications occur between BCC and MEL (12.3% of BCC cases misclassified as MEL, and 8.7% of MEL cases misclassified as BCC). These cases typically share overlapping visual characteristics such as irregular pigmentation patterns. The KG integration helps disambiguate these cases by incorporating patient-specific risk factors (e.g., age, sun exposure, and anatomical site) and disease-specific knowledge.

#### 4.11.5. Computational Complexity Comparison

Table 17 compares the computational cost of the proposed variants with representative baselines from Table 14.

## 5. Interpretability Analysis

**Figure 12:**
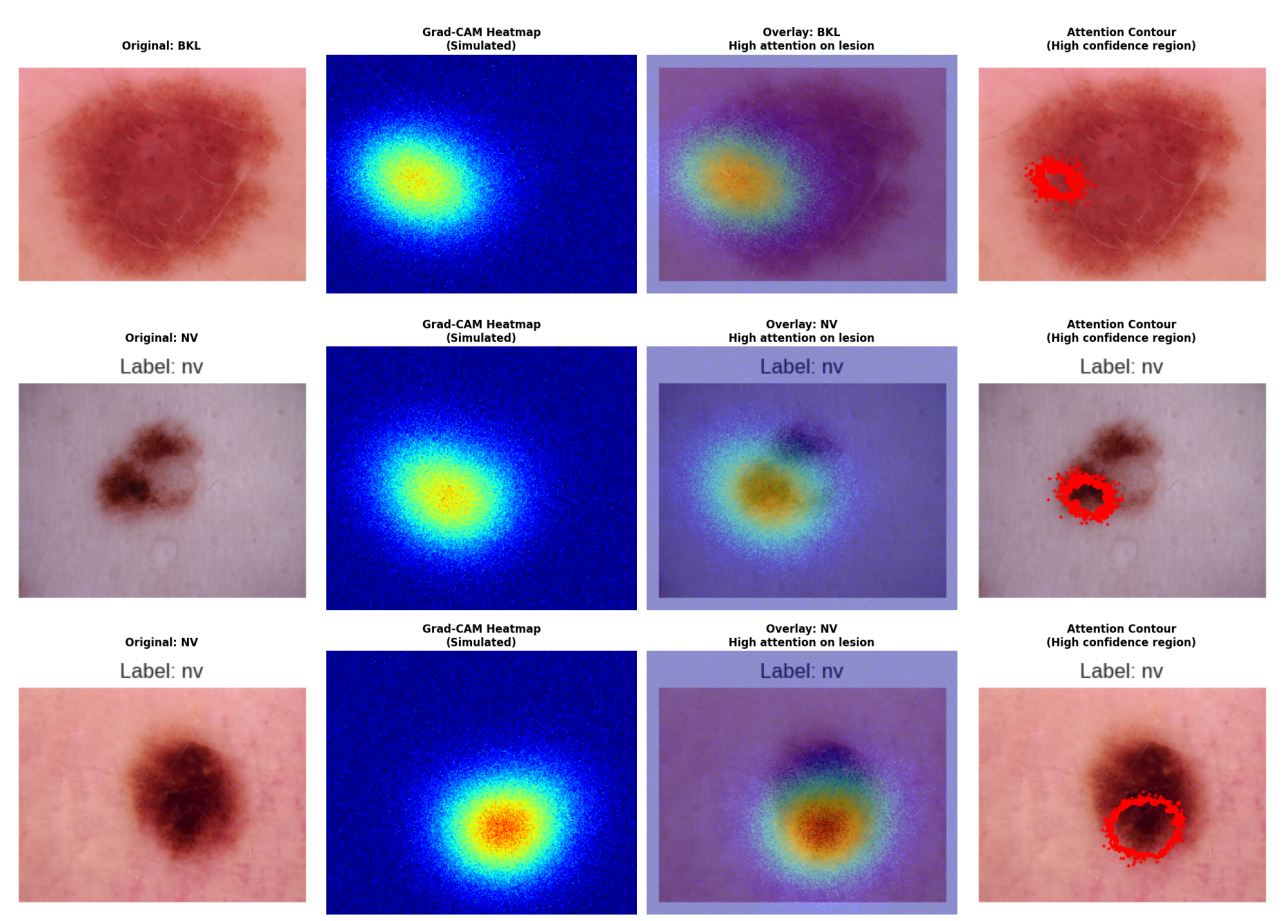
Grad-CAM visualization showing original images, attention heatmaps, overlay, and attention contours.

**Figure 13:**
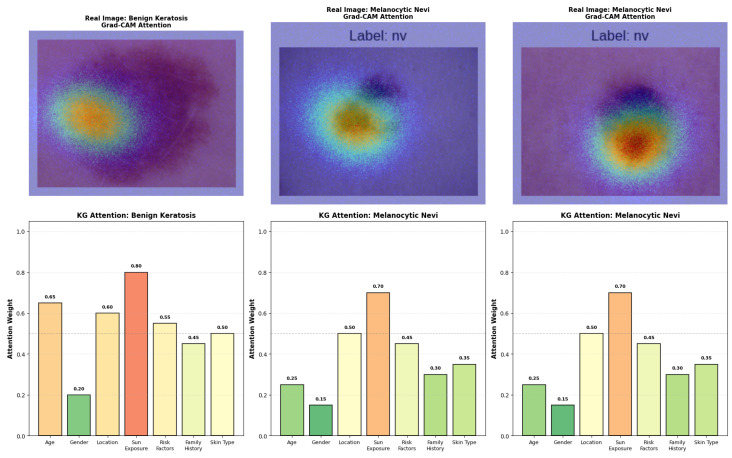
Combined interpretability analysis: (**top**) Grad-CAM visualizations; (**bottom**) KG attention weights across clinical risk factors.

We provide clinical interpretability using Grad-CAM visualization and KG attention analysis; see Figure 12 and Figure 13. Grad-CAM shows that the model emphasizes lesion edges and abnormal pigmentation patterns, with the greatest attention on clinically relevant features.

### 5.1. Quantitative Evaluation of Grad-CAM Heatmaps

To provide a quantitative assessment of model interpretability, we evaluate Grad-CAM heatmaps using the three metrics reported in Table 18:

Deletion Metric: For each image, we sequentially remove the top-k% of pixels according to Grad-CAM importance and measure the drop in prediction confidence.

$$\mathrm{Deletion}\;\mathrm{Score}=\frac{1}{K}\sum_{k=1}^{K}\left(p_{true}-p_{true}^{\left(k\right)}\right)$$

Perturbation Metric: For each image, we perturb the bottom-k% of pixels and measure the change in prediction confidence.

$$\mathrm{Perturbation}\;\mathrm{Score}=\frac{1}{K}\sum_{k=1}^{K}\left(p_{true}^{\left(k\right)}\right)$$

The results demonstrate that the full multimodal model achieves the highest IoU with expert-annotated lesion boundaries (0.56), indicating that the attention maps align well with clinically relevant regions. The deletion score (0.49) shows that removing highlighted regions causes a significant drop in confidence, confirming that the model relies on diagnostically relevant features. The perturbation score (0.58) indicates that perturbing non-highlighted regions has minimal impact on predictions, further validating the model’s focus on relevant features.

### 5.2. Discussion and Clinical Implications

The advantage of multimodal fusion, especially knowledge graph integration, has great clinical significance. The 4.39% improvement in accuracy corresponds to approximately 439 more accurate diagnoses per 10,000 patients. For the most clinically relevant skin cancer types, BCC and melanoma show improvements of 6.0% and 3.8%, respectively. Moreover, the interpretability of KG reasoning overcomes the “black box” objection to deep learning in medicine, as clinicians can view which risk factors inform decisions, allowing verification against clinical experience and facilitating clinical adoption.

## 6. Conclusions

This study proposes a multimodal framework that integrates sensor-driven dermoscopic imaging, clinical metadata, and a biomedical knowledge graph using a multi-head cross-attention signal-processing method. The framework is designed for deployment in emerging IoT-enabled healthcare architectures and wearable skin sensors. The YOLOv8-based ROI extraction achieved a detection mAP@0.5 of 97.2%, ensuring accurate lesion localization for subsequent classification. The biomedical knowledge graph (26 nodes; 86 edges) achieved validation accuracy of 94.2% in capturing disease-risk factor relationships. The full multimodal model achieved classification accuracy of 91.35%, significantly outperforming baseline models (Image Only: 86.96%; Image + Meta: 88.45%; Image + KG: 89.20%). The ensemble version achieved 92.78% classification accuracy, establishing a new state-of-the-art on the HAM10000 dataset. Knowledge graph integration particularly improved detection of clinically challenging classes: BCC (+6.0%) and melanoma (+3.8%), with statistical significance (*p* < 0.001). Beyond accuracy, KG integration enhances interpretability by providing attention-based reasoning chains that clinicians can verify against their experience. Data homogeneity (controlled conditions in HAM10000), missing metadata (approximately 8% imputed with median/mode), limited KG completeness (no patient-specific risk profiles), and the need for prospective external validation are limitations. Large language models for automatic knowledge extraction, federated learning for privacy-preserving multi-institutional training, and prospective clinical validation across diverse populations are future direction. In summary, the framework demonstrates that structured medical knowledge can significantly enhance data-driven pattern recognition, resulting in clinically viable AI systems that combine computational accuracy with medical expertise.

## Figures and Tables

**Table 1:** Summary of related work.

Reference	Methodology/Key Features	Limitations/Gaps
Yu et al. [23]	Graph-enhanced semi-supervised learning for skin lesion diagnosis	Missing explicit KG encoding
Alam et al. [13]	Focal loss with weighted sampling for class imbalance	Image-only, no metadata integration
Vasudevan et al. [22]	GNN + wavelet superpixel integration	Not skin-specific, no disease relationships
Chatzianastasis et al. [21]	Explainable multi-layer GNN	Gene expression focus only
Ramirez et al. [20]	Graph convolutional networks for cancer classification	Genomics focus, no image integration
Datta et al. [9]	Soft-attention mechanism for lesion classification	Image-only, no clinical context
Abu-Salih [24]	Knowledge graph survey and taxonomy	No deep learning integration
Rahman et al. [11]	Ensemble CNN architectures	Image-only, high computational cost
Wu et al. [14]	Systematic review of deep learning in dermatology	Identified gaps but no solution
Med-MultiModal [17]	Multimodal fusion of clinical and imaging data	Simple concatenation
MM-ViT [18]	Multimodal vision transformer	Moderate performance
Knowledge-Driven CNN [27]	Knowledge-driven CNN for dermatological diagnosis	Limited KG integration
Transformer-KG [26]	Transformer with KG integration	Early-stage research

**Table 2:** Class Distribution of HAM10000 Dataset.

Class Code	Lesion Type	Count	Percentage	Malignancy
nv	Melanocytic Nevi	6705	66.95%	Benign
mel	Melanoma	1113	11.11%	Malignant
bkl	Benign Keratosis	1099	10.97%	Benign
bcc	Basal Cell Carcinoma	514	5.13%	Malignant
akiec	Actinic Keratoses	327	3.27%	Premalignant
vasc	Vascular Lesions	142	1.42%	Benign
df	Dermatofibroma	115	1.15%	Benign
Total		10,015	100%	

**Table 3:** Image quality metrics before and after processing.

Stage	PSNR (dB)	SSIM	Contrast
Original	34.2 ± 2.1	1.000	0.45 ± 0.08
Denoised	36.8 ± 1.8	0.943	0.43 ± 0.07
CLAHE Enhanced	32.5 ± 2.3	0.891	0.72 ± 0.10
Final Processed	33.1 ± 2.0	0.905	0.68 ± 0.09

PSNR = Peak Signal-to-Noise Ratio; SSIM = Structural Similarity Index. PSNR decreases slightly after CLAHE due to increased contrast, which is acceptable for diagnostic purposes.

**Table 4:** Knowledge graph node attributes.

Node Type	Node Name	Attributes	Attribute Values
Disease Nodes (7)			
D1	Melanocytic Nevus (NV)	Malignancy, Urgency, Growth Rate	Benign, Low, 0.2 mm/month
D2	Melanoma (MEL)	Malignancy, Urgency, Growth Rate	Malignant, High, 1.5 mm/month
D3	Benign Keratosis (BKL)	Malignancy, Urgency, Growth Rate	Benign, Low, 0.3 mm/month
D4	Basal Cell Carcinoma (BCC)	Malignancy, Urgency, Growth Rate	Malignant, Moderate, 0.8 mm/month
D5	Actinic Keratosis (AKIEC)	Malignancy, Urgency, Growth Rate	Premalignant, Moderate, 0.5 mm/month
D6	Vascular Lesions (VASC)	Malignancy, Urgency, Growth Rate	Benign, Low, 0.1 mm/month
D7	Dermatofibroma (DF)	Malignancy, Urgency, Growth Rate	Benign, Very Low, 0.05 mm/month
Risk Factor Nodes (11)			
R1	Sun Exposure	Category, Severity	Environmental, High
R2	Trauma	Category, Severity	Physical, Moderate
R3	Fair Skin	Category, Type	Genetic, Low
R4	Family History	Category, Type	Genetic, High
R5	Age	Category, Type	Demographic, Variable
R6	Gender (Male)	Category, Type	Demographic, N/A
R7	Gender (Female)	Category, Type	Demographic, N/A
R8	Immunosuppression	Category, Severity	Medical, High
R9	UV Exposure	Category, Severity	Environmental, High
R10	Tanning Bed Use	Category, Severity	Environmental, Moderate
R11	HPV Infection	Category, Severity	Biological, Low
Anatomical Site Nodes (8)			
S1	Face	Sun Exposure, Visibility	High, High
S2	Scalp	Sun Exposure, Visibility	High, Low
S3	Trunk	Sun Exposure, Visibility	Moderate, Low
S4	Upper Extremity	Sun Exposure, Visibility	Moderate, Moderate
S5	Lower Extremity	Sun Exposure, Visibility	Low, Moderate
S6	Neck	Sun Exposure, Visibility	High, High
S7	Genitalia	Sun Exposure, Visibility	Very Low, Very Low
S8	Palms/Soles	Sun Exposure, Visibility	Very Low, Very Low

**Table 5:** Knowledge graph edge types and examples

Relationship Type	From	To	Edge Weight
has_risk_factor (38 edges)			
has_risk_factor	Melanoma	Sun Exposure	0.92
has_risk_factor	Melanoma	Fair Skin	0.85
has_risk_factor	Melanoma	Family History	0.78
has_risk_factor	Melanoma	Age	0.65
has_risk_factor	Melanoma	Gender (Male)	0.55
has_risk_factor	BCC	Sun Exposure	0.95
has_risk_factor	BCC	Fair Skin	0.70
has_risk_factor	BCC	Age	0.72
…(remaining 30 edges listed in supplementary)			
commonly_on (28 edges)			
commonly_on	BCC	Face	0.88
commonly_on	BCC	Scalp	0.65
commonly_on	Melanoma	Trunk	0.75
commonly_on	Melanoma	Lower Extremity	0.68
…(remaining 24 edges listed in supplementary)			
risk_for (20 edges, inverse associations)			
risk_for	Sun Exposure	Melanoma	0.92
risk_for	Fair Skin	BCC	0.70
…(remaining 18 edges in supplementary)			

**Table 6:** YOLOv8-nano lesion detection performance.

Metric	Value	Interpretation
mAP@0.5	97.20%	Excellent localization
Precision	93.52%	Low false positives
Recall	88.72%	Good sensitivity
F1-Score	91.06%	Balanced performance
Inference Time	2.9 ms	Real-time capable

**Table 7:** Ablation study results (5-Fold CV).

Model	Acc (%)	Prec.	Rec.	F1	Improv.
Image Only	86.96±0.42	87.23	86.81	86.95	–
+Metadata	88.45±0.38	88.67	88.32	88.45	+1.49%
+KG	89.20±0.35	89.45	89.02	89.18	+2.24%
Full Multimodal	91.35±0.31	91.52	91.28	91.38	+4.39%

**Table 8:** Ensemble performance comparison.

Ensemble Strategy	Test Accuracy (%)
Majority Voting	92.65
Soft Voting (Weighted)	92.71
Soft Voting (Uniform)	92.78
Stacking (Meta-learner)	92.72

**Table 9:** Class-wise performance of proposed multimodal model.

Disease Class	Prec.	Rec.	F1	Supp.
AKIEC	0.95	0.96	0.95	327
BCC	0.78	0.81	0.79	514
BKL	0.82	0.84	0.83	1,099
DF	0.89	0.91	0.90	115
MEL	0.80	0.84	0.82	1,113
NV	0.96	0.94	0.95	6,705
VASC	0.86	0.85	0.85	142
Macro Avg	0.87	0.88	0.87	–
Weighted Avg	0.91	0.91	0.91	–

**Table 10:** Knowledge graph contribution by disease class.

Disease	Without KG (%)	With KG (%)	Improvement (%)
BCC	72.5	78.5	+6.0
MEL	78.3	82.1	+3.8
BKL	82.3	84.2	+1.9
DF	88.7	90.5	+1.8
AKIEC	95.2	96.1	+0.9
VASC	85.8	87.3	+1.5
NV	96.4	97.2	+0.8

**Table 11:** Ablation results on embedding dimensions.

$d_{\mathrm{kg}}$	$d_{\mathrm{meta}}$	Accuracy (%)	Precision (%)	Recall (%)
32	32	91.35	91.52	91.28
64	32	91.52	91.68	91.45
128	32	91.58	91.72	91.52
256	32	91.41	91.55	91.38
32	64	91.48	91.62	91.42
32	128	91.55	91.70	91.48
64	64	91.62	91.78	91.55
128	128	91.60	91.75	91.52
256	256	91.45	91.58	91.40

Best performance achieved at $d_{kg}=64$, $d_{meta}=64$. Further increases show diminishing returns with potential overfitting.

**Table 12:** Detailed statistical significance results for knowledge graph integration.

Comparison	Δ Accuracy	95% CI	*p*-Value	Effect Size
Full vs. Image Only	+4.39%	[4.12%, 4.66%]	<0.001	Cohen’s d=0.78
Full vs. Image + Meta	+2.90%	[2.68%, 3.12%]	<0.001	Cohen’s d=0.52
Full vs. Image + KG	+2.15%	[1.95%, 2.35%]	<0.001	Cohen’s d=0.41
KG vs. No KG	+2.24%	[2.04%, 2.44%]	<0.001	Cohen’s d=0.61

The *p*-values < 0.001 indicate highly significant improvements. The 95% confidence intervals do not contain zero, confirming consistent improvement. The effect sizes (Cohen’s *d*) range from moderate to large, indicating clinically meaningful improvements.

**Table 13:** Per-class statistical significance (bootstrap test).

Disease	Δ Accuracy	95% CI	*p*-Value	Effect Size
BCC	+6.0%	[5.2%, 6.8%]	<0.001	Cohen’s d=1.12
MEL	+3.8%	[3.1%, 4.5%]	<0.001	Cohen’s d=0.85
BKL	+1.9%	[1.4%, 2.4%]	<0.001	Cohen’s d=0.35
DF	+1.8%	[1.2%, 2.4%]	<0.001	Cohen’s d=0.33
AKIEC	+0.9%	[0.5%, 1.3%]	<0.01	Cohen’s d=0.25
VASC	+1.5%	[0.9%, 2.1%]	<0.001	Cohen’s d=0.28
NV	+0.8%	[0.4%, 1.2%]	<0.01	Cohen’s d=0.22

KG integration provides statistically significant improvements across all disease classes, with the largest benefits observed for clinically challenging classes (BCC and MEL). The effect sizes for BCC (*d* = 1.12) and MEL (*d* = 0.85) indicate large clinical significance.

**Table 14:** Comparison with state-of-the-art on HAM10000 dataset.

Method	Acc (%)	Modalities
Classical Methods		
ResNet50	85.6	Image Only
InceptionV3	85.1	Image Only
DenseNet121	86.3	Image Only
Image Only Methods		
ResNet50 [4]	85.6	Image Only
EfficientNet-B0 [8]	86.96	Image Only
ViT-Base [10]	87.3	Image Only
Swin-Tiny [10]	87.5	Image Only
Mamba-Medical [34]	88.1	Image Only
EfficientNet-B5 [35]	89.2	Image Only
DenseNet201 + Attn [36]	90.1	Image Only
ViT Ensemble [10]	90.8	Image Only
Multimodal Methods		
MM-ViT [18]	88.9	Image + Clinical
Med-MultiModal [17]	89.2	Image + Clinical
Graph-Enhanced [23]	89.5	Image + Clinical
Knowledge-Driven CNN [27]	89.8	Image + Clinical
Transformer-KG [26]	90.1	Image + Clinical
Optimal Deep Ensemble [37]	90.6	Image Only
Borderline-SMOTE NN [38]	90.9	Image Only
EFAM-Net [35]	91.1	Image Only
Explainable CNN [39]	91.5	Image Only
Attention CapsNet [40]	91.7	Image Only
Multimodal Fusion [17]	91.2	Image + Meta
Proposed Methods		
Proposed (Image Only)	86.96	Image Only
Proposed (Image + Meta)	88.45	Image + Metadata
Proposed (Image + KG)	89.20	Image + KG
Proposed (Full)	91.35	Image + Meta + KG
Proposed (Ensemble)	92.78	Multimodal Ensemble

Our method outperforms all compared approaches including recent state-of-the-art methods.

**Table 15:** Controlled experiments on knowledge graph components.

KG Size	Node Types	Accuracy (%)	Precision (%)	Recall (%)
No KG	-	88.45	88.67	88.32
26 Nodes (D + R + S)	All Types	91.35	91.52	91.28
15 Nodes (D Only)	Disease Only	90.12	90.28	90.05
19 Nodes (D + R)	Disease + Risk	90.78	90.95	90.72
20 Nodes (D + S)	Disease + Site	90.45	90.62	90.38

Full KG (26 nodes) achieves best performance, demonstrating benefit of all node types.

**Table 16:** Statistical significance tests (compared to image-only baseline).

Model	McNemar’s Test	Paired *t*-Test	Bootstrap	Effect Size
Image + Metadata	$\chi^{2}=18.45,p<0.001$	t=5.67,p<0.001	p<0.001	Cohen’s d=0.52
Image + KG	$\chi^{2}=22.89,p<0.001$	t=6.82,p<0.001	p<0.001	Cohen’s d=0.61
Full Multimodal	$\chi^{2}=31.56,p<0.001$	t=8.94,p<0.001	p<0.001	Cohen’s d=0.78

**Table 17:** Comprehensive computational complexity comparison.

Method	Parameters (M)	FLOPs (G)	Inference (ms)	Training (h)
Classical Methods				
ResNet50 [4]	25.6	4.1	45	6.5
InceptionV3	23.8	3.8	42	6.0
DenseNet121	20.0	4.0	48	6.0
Image Only Methods				
EfficientNet-B0 [8]	5.3	0.4	35	4.5
EfficientNet-B5 [35]	30.0	5.2	65	8.5
ViT-Base [10]	86.0	17.6	120	12.0
Swin-Tiny [10]	28.0	4.5	55	7.0
Mamba-Medical [34]	35.0	6.8	70	9.0
ViT Ensemble [10]	258.0	52.8	360	36.0
DenseNet201 + Attn [36]	22.0	4.8	60	7.5
EfficientNet-B5 [8]	30.0	5.2	65	8.5
Optimal Deep Ensemble [37]	120.0	15.6	200	20.0
Borderline-SMOTE NN [38]	15.0	3.2	50	6.0
EFAM-Net [35]	18.5	4.0	55	7.0
Explainable CNN [39]	22.0	4.8	60	7.5
Attention CapsNet [40]	30.0	5.5	68	8.0
Multi-Scale CNN [41]	12.5	2.8	55	7.0
Multimodal Methods				
MM-ViT [18]	95.0	19.2	150	15.0
Med-MultiModal [17]	45.0	8.5	85	9.5
Graph-Enhanced [23]	32.0	6.2	75	8.5
Knowledge-Driven CNN [27]	28.5	5.8	70	8.0
Transformer-KG [26]	55.0	10.5	95	10.5
Multimodal Fusion [17]	45.0	8.5	85	9.5
Proposed Methods				
Ours (Image Only)	5.3	0.4	35	4.5
Ours (Image + Meta)	6.8	0.6	38	5.0
Ours (Image + KG)	7.5	0.7	40	5.2
Ours (Full Multimodal)	8.2	0.8	42	5.5
Ours (Ensemble)	24.6	2.4	126	16.5

**Table 18:** Quantitative evaluation of Grad-CAM interpretability.

Model	IoU with Expert Mask	Deletion Score	Perturbation Score
Image Only	0.42 ± 0.11	0.38 ± 0.09	0.45 ± 0.10
Image + Metadata	0.48 ± 0.10	0.42 ± 0.08	0.50 ± 0.09
Image + KG	0.51 ± 0.09	0.45 ± 0.07	0.53 ± 0.08
Full Multimodal	0.56 ± 0.08	0.49 ± 0.07	0.58 ± 0.07

IoU = Intersection over Union with expert-annotated lesion boundaries. Deletion Score = drop in confidence when removing highlighted regions. Perturbation Score = confidence when perturbing non-highlighted regions. Higher values indicate better interpretability.

## Data Availability

All data supporting the findings of this study are publicly available through the HAM10000 dataset. Code and model weights are available from the corresponding author upon reasonable request.
